# An Introduction to Survival Statistics: Kaplan-Meier Analysis

**DOI:** 10.6004/jadpro.2016.7.1.8

**Published:** 2016-01-01

**Authors:** William N. Dudley,1, Rita Wickham,2, NICHOLAS COOMBS,3

**Affiliations:** 1University of North Carolina Greensboro, School of Health and Human Sciences, Department of Public Health Education, Greensboro, North Carolina; Piedmont Research Strategies, Inc., 2Rush University College of Nursing, Chicago, Illinois; RSW Consulting, LLC, 3Billings Clinic, Center for Clinical Translational Research, Billings, Montana

Studies of how patients respond to treatment over time are fundamentally important to understanding how therapies influence quality of life and progression of disease during survivorship. When investigators examine change over time in continuous variables (e.g., patient self-reports of pain, fatigue, or nausea) in the same individuals, repeated measures are typically analyzed using analysis of variance (ANOVA) or perhaps latent growth curve modeling (Brant et al., 2011; Dudley, McGuire, Peterson, & Wong, 2009). Other studies—particularly those that compare the long-term effects of new drugs or other therapeutic regimens to some "standard" therapy—focus on time to *binary* (yes/no) disease-related events of interest, such as death (time to event). Such studies are particularly apropos to generating improvements in cancer therapies, in which new treatments are compared to "standard" regimens, and are shown or disproved to extend progression-free survival (PFS), time to progression, or overall survival (OS) in patients with a particular cancer.

Time-to-event studies typically employ two closely related statistical approaches, Kaplan-Meier (K-M) analysis and Cox proportional hazards model analysis (sometimes abbreviated as proportional hazards model or Cox model). K-M is a univariate approach, while Cox analysis is multivariable. Both use many familiar aspects of parametric and nonparametric statistical techniques (e.g., independent and dependent variables, null hypothesis testing, and confidence intervals). On the other hand, survival analyses employ other analytical techniques, terms, and computations that some oncology advanced practitioners (APs) may be less familiar with.

No published research that addressed oncology APs’ knowledge and ability to interpret statistical tests was found, but a study of medical residents examined their knowledge within the context of statistical procedures used in medical studies (Windish, Huot, & Green, 2007). Results proved that there was a mismatch between statistical procedures used and these clinicians’ understanding, and therefore the ability to judge the quality and veracity of published research. That is, more than 81% correctly interpreted relative risk, but only 10.5% understood K-M, and 11.9% could interpret the 95% confidence interval (CI) and statistical significance. Given that APs’ knowledge deficits may be somewhat similar (consider your own understanding of these concepts), this article will succinctly describe and illustrate K-M analysis.

## KAPLAN-MEIER ANALYSIS

Kaplan and Meier (1958) first described the approach and formulas for the statistical procedure that took their name in their seminal paper, *Nonparametric Estimation From Incomplete Observations*. They described the term "death," which could be used metaphorically to represent any *potential* event subject to random sampling, particularly when complete observations of all members of a random sample cannot be made. Incomplete observations often occur because contact with some sample members is lost before the event, some other intervening variable affects the *event*, or insufficient time has passed to observe the event in all sample members. Any of these cases would result in a participant being *censored*, as discussed further below. An event is a binary variable that can only have a *yes* or *no* value (e.g., death, hospital discharge or readmission, heart attack, recovery from an infection, or relapse from smoking cessation, etc.). K-M analyses are not unique to medical studies; they are used by researchers in other disciplines to study time to particular events.

**Survival Analyses**

Survival analyses are statistical methods used to examine changes over time to a specified event. K-M is the most frequent survival analysis method used in randomized (phase III and some phase II) medical clinical trials in which the following criteria are met:

- Patients are randomly assigned to different treatment arms;
- All patients do not enter the study at the same time;
- Patients drop out of or are lost from the study at different time intervals after entering the study; and
- The outcome variable of interest may or may not occur during the study observation period (Rich et al., 2010).

K-M can calculate how long after starting a particular treatment that the studied event (e.g., death, disease progression, etc.) occurred for individuals who were not otherwise lost to the sample—or until the study has ended (Peto et al., 1977; Rich et al., 2010).

**Underlying Concepts and Terms**

Understanding studies analyzed with K-M requires appreciation of associated concepts, terms, assumptions, and methods. Other important concepts are the "rules" for the study and K-M analysis set before the study is implemented. These include the conditions under which a study will be stopped early, stopping boundaries, how to deal with missing data, and the number and points of data analyses.

One important factor is that patients enter clinical trials and are eliminated from the sample (and data analysis) at different times: when a study opens, as accrual continues, for a predetermined period, or until a desired sample size is reached, as patients die (or experience another event of interest) or are lost from the sample for another reason. When patients are lost from a K-M study for any reason, they are considered to be censored. Being censored does not have any negative connotation; it is merely part of the language of K-M.

Censoring is a major difference between K-M and more traditional parametric analyses, in that researchers must adjust the data at each point where one or more patients are lost from the study for any reason to take censored cases into account (Rich et al., 2010). Sample members become censored when investigators cannot determine if or when a subject ultimately experiences the negative event, and it can occur during (when the subject experiences the event or otherwise drops out or is lost from the study) or at the end of the study (right censoring of all remaining subjects because no further data will be collected). Important assumptions are that censored patients have the same likelihood of survival as those continuing in the study (an assumption not easily testable), and that survival probabilities are the same whether individuals enter a study early or late (can be examined with split-half analysis; Jager et al. 2008). Censored patients are included in probability estimates of the event to the evaluation point preceding their censoring, adding a maximum amount of data, but are eliminated from subsequent analyses ([Bibr A2]Blagoev, Wilkerson, & Fojo, 2012).

Missing data is a problem that can potentially bias data analysis and statistics. One important way to deal with this is to use the intent-to-treat strategy, which includes all patients who entered the study in the sample denominator and requires patient follow-up and data collection whenever possible (Shih, 2002). Another strategy that can help address this problem is to track the numbers of patients in each arm who withdraw and reasons for withdrawal and to include this information in research reports.

**Figure 1:**
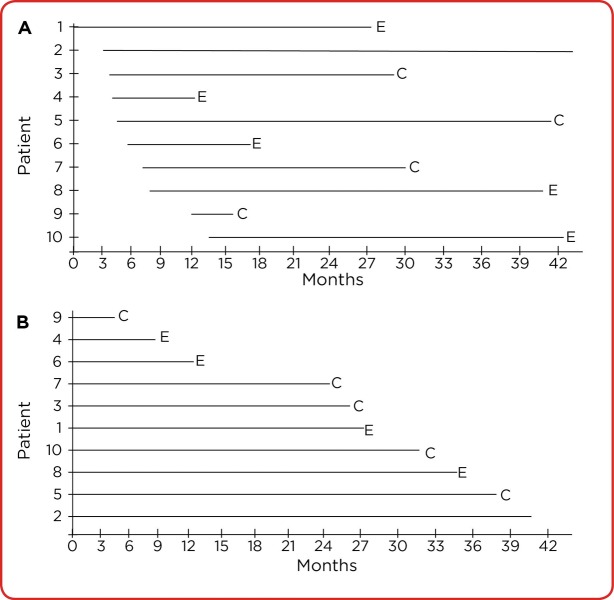
(A) Hypothetical first patients assigned to arm A as they sequentially enter the study. Each patient’s serial time (on study) ends with an E (for death in this example) or a C (for censored). If the data analysis is completed at the end of the period marked by the right border of the x-axis, all patients who have not died or already been censored are censored at that point. (B) The same hypothetical patients are arranged from shortest to longest serial time before K-M analysis, allowing us to see the initial intervals that will be graphed on the horizontal (time) axis.

A way to envision these concepts is to consider a hypothetical trial, and the first 10 consenting patients randomized to arm A shown in Figure 1A. We can appreciate the sequential order that patients in the cohort entered the study, and whether they experienced the event (E) or were censored (C). We cannot determine how long each patient remained on study (his or her serial time) before E or C occurred, but this might be a brief or extended period. Some study participants do not experience the study event, and others are dropped or are withdrawn from the study for one or more reasons. For instance, since data collection has not yet ended, patient 2 has not experienced the outcome and has not yet been censored, as the serial line is continuing (if this were the endpoint for data collection, all remaining patients become censored). Before data analysis, all patients in each cohort are first arranged from the shortest to longest serial time (time on study) and are analyzed as if they all began the study at the same time point, as shown in Figure 1B. In this representation, it is easier to see that patients have varying serial times to the event or to becoming censored (Jager, van Dijk, Zoccali, & Dekker, 2008; Rich et al., 2010).

In addition, rules for boundaries to stop a study early, and the number of endpoints of planned data analyses, should be explicit before a study is implemented. A predetermined stopping boundary is a method to determine if a study can be stopped early—for instance, when the primary outcome variable has been reached (Pocock, 2005). A stopping boundary must be stringent (e.g., have a small p value) to support meaningful clinical differences in treatments, which is suggested to be .01 to confirm clinical benefit. This is crucial to legitimately support clinically relevant, evidence-based practice; to achieve an adequate sample size within the intended duration of a trial; to positively change particular therapies; to meet the goals of researchers and regulators to conduct scientifically rigorous studies; and to disseminate data supporting therapy advances as rapidly as possible (Zannad et al., 2012). When a highly statistically significant clinical trial benefit is confirmed and leads to early stopping, the researchers have an ethical responsibility to offer the better treatment to patients in the less effective treatment arm (considering that adverse effects and treatment burdens do not outweigh benefits). For example, costs of therapy may be a burdensome limitation for some patients because of insurance reimbursement policies.

**Interpreting a Kaplan-Meier Plot**

The statistical output for a K-M analysis offers a visual representation of predicted survival curves (i.e., from not experiencing the event of interest) of two or more groups. It is not a smooth curve or line, but it has a distinctive monotonic (one-direction) stair-step appearance. For any K-M estimator, the horizontal x-axis represents the time variable expressed in a linear fashion (i.e., weeks, months, years, etc.). All patients start at the top (1.0 or 100%) of the y-axis, which indicates the sample proportion that has not experienced the studied event. Each horizontal line (except for the first) begins and ends with the occurrence of the event in two subsequent patients in a treatment arm (Jager et al., 2008; Rich et al., 2010). Only the event influences the duration of a particular interval, whereas censored patients are usually indicated by tick marks (or dots) along the interval in which they were censored.

In cancer clinical trials, negative events (e.g., PFS or OS) result in a left to right descending pattern as patients no longer "survive" the event: They experience disease progression or death. Survival curves can actually go down or up to show the same information over time; downward plots display patients who have not experienced the event, whereas upward plots illustrate the cumulative patients who did experience the event (Pocock, Clayton, & Altman, 2002).

**Figure 2:**
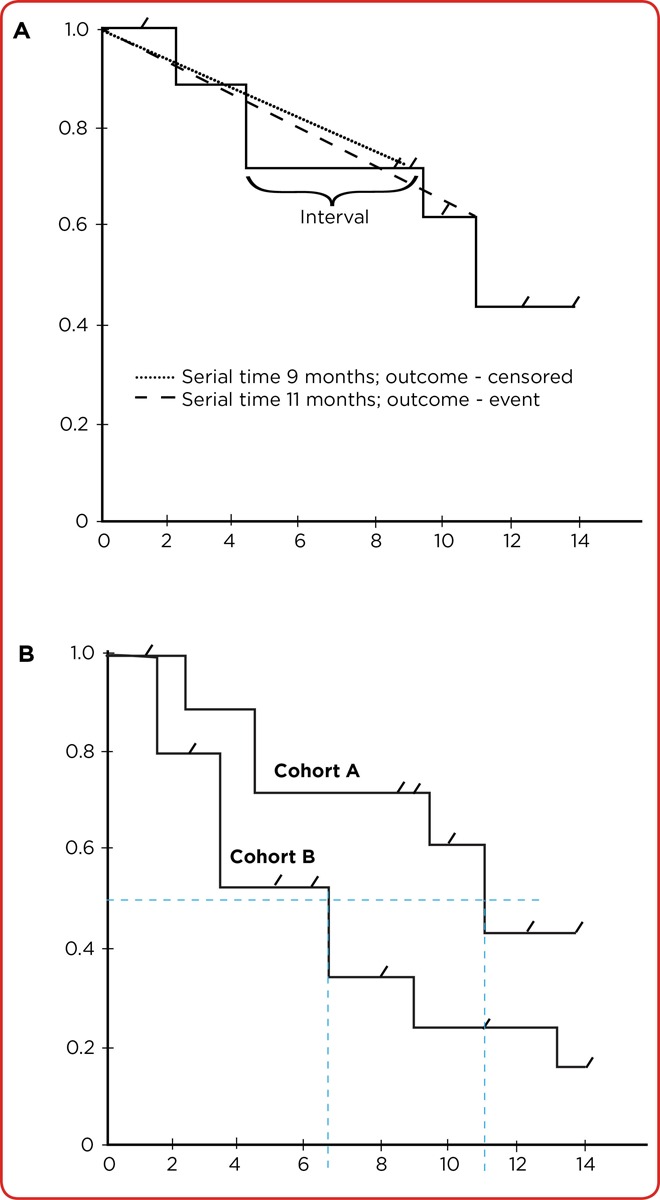
(A) A hypothetical Kaplan-Meier curve of one cohort (arm). Each horizontal portion is the interval between the studied event between one and the next subject in that arm. Only the event influences the interval length, whereas tick marks indicate censored subjects. (B) Median survival (from experiencing the studied event) can be estimated in both arms by drawing a line on the y-axis at 0.5 (50%). Locating the point at which each intersects 0.5 shows median survival is approximately 6.5 months in cohort B and 11 months in cohort A.

The length of each horizontal line represents the survival duration for that interval, and all survival estimates to a given point represent the cumulative probability of surviving to that time. Intervals are not identical, and a strength of the K-M plot is that it can manage varying interval lengths (Rich et al., 2010). Figure 2A shows how each study interval (after the first) begins with the studied event in one patient and ends with the event in the next patient in that cohort. This leads to the K-M plot looking like a series of downward steps. The probability of surviving an interval is related to the number of patients in that interval: Both the numerator and the denominator decrease by the number of patients who experienced the event plus those who were censored. Each of these probabilities contributes to the subsequent and final probability of not experiencing the event (e.g., progression or death).

The "steps" of the K-M plot provide the visual representation of individuals who have or have not experienced the event. We can look at the K-M plot in Figure 2A and calculate predicted survival for the first interval. Assuming the original sample had 10 patients, if we did not consider the censored patient, the estimated survival at this point (the first drop) would be 9/10 (90%). However, this is actually 8/9 (88.8%). Each interval is assumed to be independent, and each affects the subsequent interval. The vertical lines connect each interval and represent the decrease in likelihood of having experienced the event at that point. In Figure 2A, at 2.5 months after starting the study, the chance of not experiencing the event is 88.8%. It is important to understand that this example only serves to illustrate a K-M "curve" in a very simple fashion. Very small samples are prone to error and are less valid and reliable than large samples. If patients were still accruing to the study, analysis would occur at a later, more reasonable time to judge treatment efficacy.

K-M curves with many small steps have larger sample sizes, while those with large steps usually have a limited number of subjects and are thus less accurate (Rich et al., 2010). "Drops" can be seen in the curve at variable intervals, and in studies with long observation times, bigger decreases toward the right side of the plot are seen because later events are larger fractions of the probability estimate for the remaining cohort (Blagoev et al., 2012). Similarly, few surviving patients at the right side of the K-M curve mean less accurate survival estimates and greater uncertainty compared to when many patients are in a study. It has been recommended to halt estimations of survival curves when the proportion of patients who have not experienced the event becomes unduly small, perhaps when only 10% to 20% of an original large sample or fewer than 10 patients in a small study are still being followed (Bollschweiler, 2003; Pocock et al., 2002).

If we look at the same hypothetical K-M plot with both treatment arms included (Figure 2B), we can see that both curves pass through the 50th percentile point. If a curve passes through 50%, the reader can quickly estimate median survival for patients in that treatment arm by drawing a vertical line from where the curve crosses the 50% to the x (time) axis and comparing median survival if both curves pass through the 50% point. We can see in the hypothetical example that median survival (50% of patients would be estimated to be surviving) is about 11 months for treatment A and 6.5 months for treatment B. Median survival is reported in most studies because survival times are usually skewed, and the median is a better measure of centrality than the mean. Furthermore, there is no way to know if or when patients who are alive and not censored at the end of a study will experience the event of interest, so a mean cannot be calculated (Jager et al., 2008). The reader can also see that the curves appear to have separated. Again, this is not a realistic K-M example, but if this separation were consistent over time, it would give us confidence about real treatment differences.

K-M estimates are most commonly reported with the log-rank test or with hazard ratios. The log-rank test calculates chi-squares (𝝌₂) for each event time, which are summed to calculate an ultimate chi-square for each arm (Jager et al., 2008; Rich et al., 2010). Log-rank results compare the full curves of each group and generate a significance level (*p* value; Rich et al., 2010). The log-rank test allows between-group comparisons of survival estimates but not the size of a potential difference or of confounding variables such as age.

Hazard ratios quantify the opposite likelihood: that the "hazardous" event will occur during study intervals (Blagoev et al., 2012), and are similarly calculated by summing 𝝌₂ for each event, providing the final observed and expected numbers for the full K-M curve (Rich et al., 2010). In the simplest terms, a hazard ratio expresses the chance (or hazard) of the events occurring in the treatment arm as a ratio of the events occurring in the control group. A hazard ratio has no dimensions and by itself provides only information about the uniformity and reliability of the data (Blagoev et al., 2012). Hazard ratios change over time and are reflected in the slope of the K-M plot. Reported hazard ratios assume that the differences between groups are a constant distance apart (i.e., the K-M survival curves) and are proportional. If this assumption is not met, a reported hazard ratio is irrelevant. A hazard ratio of greater than 1 or less than 1 means that survival was better in one of the groups (Spruance, Reid, Grace, & Samore, 2004).

## DISCUSSION: CLEOPATRA ANALYSIS

The Clinical Evaluation of Pertuzumab and Trastuzumab (CLEOPATRA) trial, a randomized, double-blind, multinational phase III study, accrued 808 patients in the intent-to-treat population over 29 months (2008 to 2010). Study findings were published in three articles (Baselga et al., 2012; Swain et al., 2013, 2015). The study timeline is briefly summarized in Table 1. The CLEOPATRA study was sponsored by a pharmaceutical company that, in partnership with the senior academic authors, collected and analyzed the data (no independent statistician was reported). An overview of the CLEOPATRA trial can be found in the article by Karen Herold.

**Table 1:**
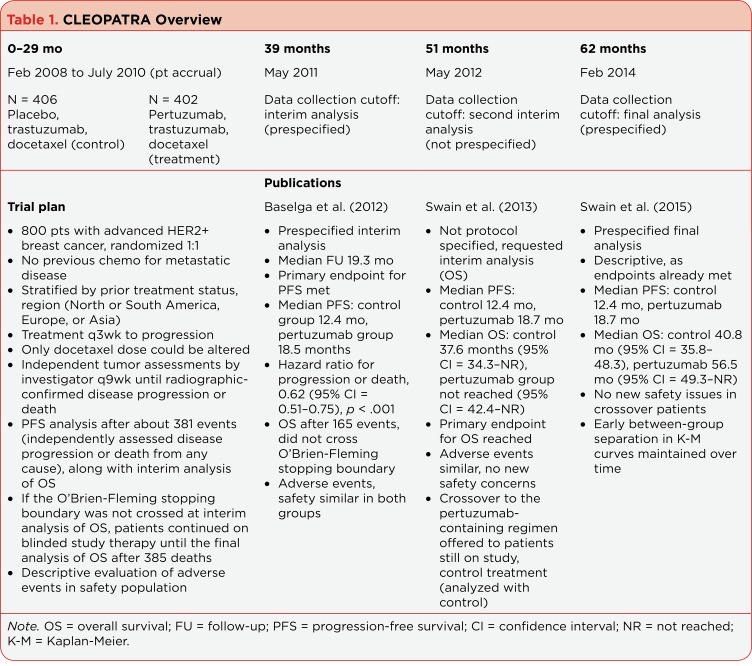
CLEOPATRA Overview

The study was planned with two prespecified K-M analyses: an interim one and a final one done after 381 events of disease progression or death from any cause (Baselga et al., 2012). Random assignment resulted in 406 patients in the control group (placebo + trastuzumab + docetaxel) and 402 patients in the pertuzumab group (pertuzumab + trastuzumab + docetaxel); treatment was planned for every 3 weeks to progression. Only the dose of docetaxel could be altered. Eligible patients had locally recurrent, unresectable, or metastatic (no central nervous system spread) HER2-positive breast cancer. Independent tumor assessments were done every 9 weeks until disease progression or death. The primary endpoint was PFS, defined as "radiographic confirmation of disease progression by Response Evaluation Criteria in Solid Tumors (RECIST) guidelines or death from any cause within 18 weeks after the last independent tumor assessment." The study employed the common practice of using date of the radiographic confirmation as the date of disease progression, which most likely occurred somewhere between the 9-week tumor assessment intervals. This would increase the duration of PFS and introduce bias into calculated median survival (Panageas, Ben-Porat, Dickler, Chapman, & Schrag, 2007).

In accordance with the requirements for a trustworthy K-M trial, a single, prespecified interim analysis of the primary study outcome of PFS after 381 patients experienced progression was planned (Baselga et al., 2012). This was calculated to give the study an 80% power to detect a 33% improvement in median PFS in the pertuzumab group. PFS is frequently the primary endpoint, particularly as new targeted therapies become available (Panageas et al., 2007). Progression-free survival is a desirable outcome because it is not influenced by later-line therapies and can be measured earlier than OS. This reduces new drug development time and more rapidly brings effective agents to market.

In CLEOPATRA, K–M was used to estimate the independently assessed median PFS in each group, log-rank test to compare PFS between the two groups, and Cox proportional-hazards model to estimate the hazard ratio and 95% CIs. Analysis of OS was planned to be done after 385 patients had died if the stopping boundary had not been crossed (Baselga et al., 2012). Other secondary endpoints objective response (OR) rate and safety would be analyzed at this point.

In this analysis, 80.2% of patients in the pertuzumab group and 69.3% in the control group experienced an OR (Baselga et al., 2012). Median PFS was 18.5 months in the pertuzumab group and 12.4 months in the control group. This exceeded the study hypothesis that median PFS would be 33% greater in the pertuzumab than in the control group. The hazard ratio for progression or death was 0.62 (95% CI = 0.51–0.75), *p* < .001 in favor of pertuzumab.

Similar to odds ratios and relative risk, a hazard ratio is interpreted as such: Those in the treatment (pertuzumab) group experienced death at a rate 38% less than those in the control group. This decrease could be as great as 49% or as little as 25% with 95% confidence. With this interval ranged less than and not including the value of one, we would conclude the hazard ratio is both *protective* and *statistically significant*. Interim analysis of OS was done after 165 events: 96 deaths in the control group and 69 in the pertuzumab group. The data showed a "strong trend toward a survival benefit" with pertuzumab and the hazard ratio was 0.64 (95% CI = 0.47–0.88; *p* = .005), which the authors stated was not statistically significant (recall from the earlier discussion that the p value should be ≤ .01). The number of patients censored and the reasons for censoring were not included in this paper, possibly because both disease progression and death were included in the definition of not meeting PFS.

After another year of follow-up, a second *unplanned* interim analysis of CLEOPATRA was done because European health authorities requested more information about OS of study patients (Swain et al., 2013). By that time, 267 deaths—154 in the control group and 113 in the pertuzumab group (69% of prespeciﬁed total for OS)—had occurred. The stopping boundary for each interim analysis was preset to use the O’Brien-Fleming approach to deal with multiple data analyses ("multiple looks"), and the stopping boundary was crossed.

Multiple looks, or data-dependent stopping, are used to find evidence of a significantly large treatment difference to end a study earlier than originally planned. The major problem with this is the likelihood of type I error (falsely rejecting the null hypothesis that there is no difference between treatments) increases with each interim analysis, so unplanned interim analyses are discouraged. A second problem is "multiple outcomes," which occurs when a study that focuses on one outcome necessarily focuses on other outcomes that are likely interdependent and not independent. For instance, the definition of PFS includes disease progression or death, which overlaps with OS. In addition, most patients probably died from their disease, but deaths could be related to other factors.

Swain and colleagues (2013) correctly recognized the importance of not increasing the risk for type I error in the analysis of OS. They amended the protocol again to apply the O’Brien-Fleming stopping boundary, defined as a significance level of ≤ .0138 and a hazard ratio of ≤ 0.739. More patients in the control group died than did those in the pertuzumab cohort, 154 of 406 (38%) and 113 of 402 (28%), respectively. The hazard ratio of 0.66 (95% CI = 0.52−0.84, *p* = .0008) crossed the preset O’Brien-Fleming stopping boundary, leading the authors to conclude there was a statistically significant OS benefit for patients who had received pertuzumab in addition to trastuzumab plus docetaxel.

The final CLEOPATRA article was descriptive and updated OS and PFS (Swain et al., 2015). This was essentially the icing on the cake because significant benefit for adding pertuzumab to trastuzumab plus docetaxel had been established in the reported second interim analysis (Swain et al., 2013). The definition of PFS was changed to "the time from randomization to documented radiographic evidence of progression" (no mention of death).

**Figure 3:**
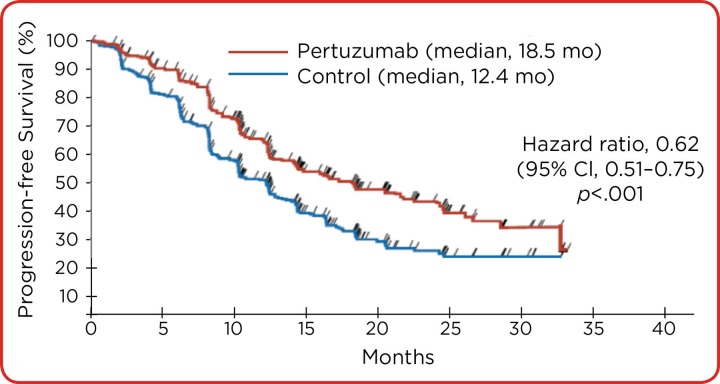
Kaplan-Meier estimates of progression- free survival in patients in the intention to- treat population in the CLEOPATRA trial. Tick marks designate the times of events. This highlights the importance of carefully reading legends, particularly in Kaplan-Meier curves in which tick marks or dots usually indicate censored individuals. Adapted from Baselga et al. (2012).

**Figure 4:**
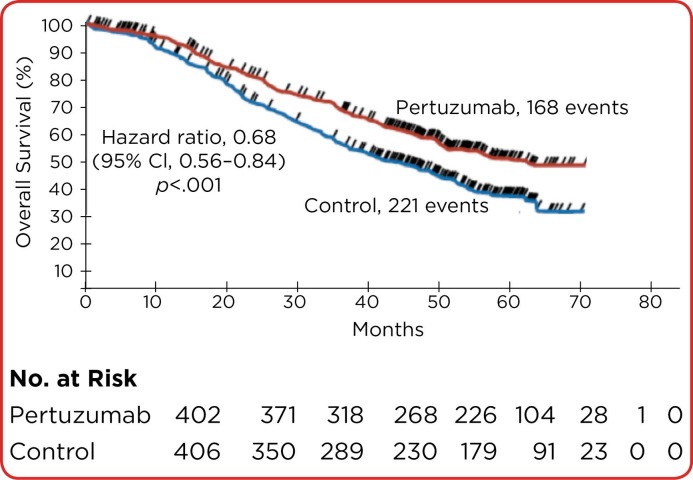
Kaplan-Meier estimates of overall survival in the intention-to-treat population in the CLEOPATRA trial. In this curve, tick marks indicate censored patients. Because this curve shows overall survival, censored patients most likely experienced progressive disease, and some of the early ones were probably docetaxel-related toxicity. If we look at the plot and estimate overall survival, our calculations will be close to what was found in the statistical analysis (56.5 months in the pertuzumab group and 30.8 months in the control group). Note that the number at risk decreases as the curve moves to the right, and most patients have been censored or died. According to recommendations, analysis after 60 months would not be recommended because of decreased accuracy. Adapted from Swain et al. (2015).

Patients who were alive or lost to follow-up were censored at the last date they were known to be alive, which is what we would expect in K-M analysis (Swain et al., 2015). The most common reason for censoring was disease progression, followed distantly by life-threatening adverse treatment-related events (see Table 2). In addition to changing definitions for the final analysis, K-M curves were handled differently. That is, in Baselga et al. (2012), the PFS K-M plot indicates all points at which events took place as tick marks on the plot (Figure 3). The reason for this was not given, but it illustrates the importance of reading figure legends. On the other hand, Swain and colleagues (2015) showed the K-M curve we would expect, with tick marks showing the time points at which patients were censored (Figure 4).

**Table 2:**
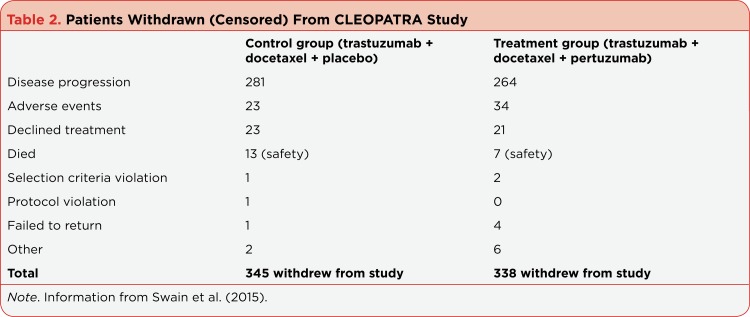
Patients Withdrawn (Censored) From CLEOPATRA Study

A total of 168 (41.8%) in the pertuzumab group and 221 (54.4%) in the control group had died by the time of the final report (Swain et al., 2015). As expected, the hazard ratio favored the pertuzumab cohort (0.68; 95% CI = 0.56–0.84; *p* < .001). Median OS in the pertuzumab group was 56.5 months (95% CI = 49.3 mo–not reached) and 40.8 months (95% CI = 35.8–48.3 mo) in the control group: a difference of 15.7 months. Estimates of OS shown in Table 3 illustrate the fact that the likelihood of being alive was greater at 1, 2, 3, and 4 years for patients receiving pertuzumab than those in the control group (Swain et al., 2015). The reported CIs overlapped only at year 1, meaning that these values were within the bounds of random chance (Pocock et al., 2002).

**Table 3:**
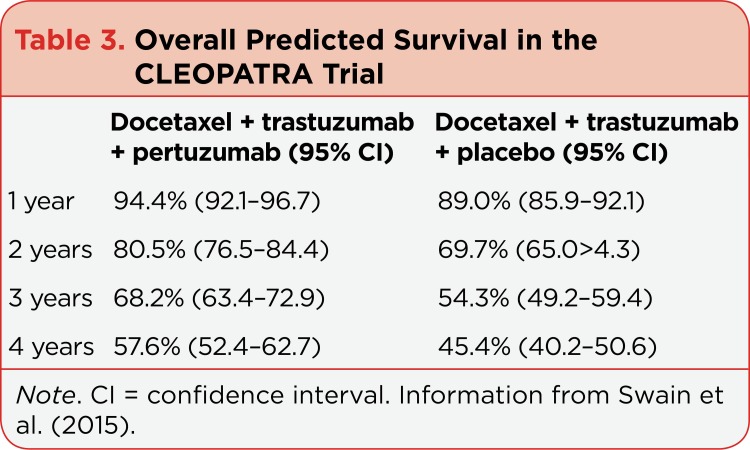
Overall Predicted Survival in the CLEOPATRA Trial

## CONCLUSION

In sum, K-M analyses of the CLEOPATRA study met the expectations of the statistical technique and addressed potential limitations. For instance, the issue of multiple looks was correctly addressed by making the p value more stringent. The authors also included important measures of statistical uncertainty—confidence intervals—that support the CLEOPATRA conclusions and give readers confidence in the research reports.
